# Validity of prenatal AUDIT screening for alcohol disorders – a Nationwide Swedish register study

**DOI:** 10.48101/ujms.v129.10770

**Published:** 2024-11-22

**Authors:** Susanne Hesselman, Joline Asp, Ulrika Pellas, Susanne Lager, Anna Wikman

**Affiliations:** a Department of Women’s and Children’s Health, Uppsala University, Uppsala, Sweden; b Center for Clinical Research Dalarna, Falun, Sweden; c Centre for Clinical Research Sörmland, Uppsala University, Eskilstuna, Sweden; d Department of Research and Education, Region Dalarna, Falun, Sweden

**Keywords:** Alcohol-related disorders, pregnancy, prenatal care, screening, diagnostic techniques and procedures

## Abstract

**Objective:**

This study aims to assess the external validity of the Alcohol Use Disorders Identification Test (AUDIT) in Swedish prenatal care as an indicator for alcohol-addiction disorders, and to characterize women with mismatched information in healthcare registers

**Design:**

This study was designed as a National register-based study.

**Setting:**

Sweden

**Participants:**

The study sample included 739,735 pregnancies over the period 2014–2020.

**Methods:**

Prospectively collected prenatal AUDIT screening in the *Swedish Pregnancy register* was linked to national health databases through individual identification number. The AUDIT score was dichotomized into < 6 points (low-risk use) and ≥ 6 points (hazardous use). Alcohol addiction disorders were defined by a diagnostic code in *The Swedish National Patient Register* or drugs dispensed for alcohol dependence in the *Swedish Prescribed Drug Register*.

**Primary Outcome Measures:**

The diagnostic properties of AUDIT were assessed based on sensitivity, specificity, positive predictive value (PPV), negative predictive value (NPV), positive likelihood ratio (LR+), negative likelihood ratio (LR-), and accuracy (proportion of true positive and true negative) for an AUDIT score of ≥ 6 points for alcohol disorders. Women with mismatched information in the register were characterized and assessed by multinominal logistic regression, using women with matched information in the registers for reference.

**Results:**

An alcohol-related disorder was recorded in 3.1%, while 25,770 (3.5%) had an AUDIT point ≥ 6. The diagnostic accuracy of the AUDIT ≥ 6 points for detection of an alcohol related disorder during a year prior to pregnancy was 95.7% (95% confidence interval [CI]: 95.7, 95.8), with a positive LR of 8.03 (95% CI: 7.5, 8.6). The sensitivity for detecting a pre-pregnancy alcohol related disorder was 33.0% (95% CI: 30.9, 35.1). Being young, nulliparous, of low education, and of Swedish origin increased the likelihood of being misclassified with the AUDIT. Prior psychiatric care was associated with false negatives, especially for women with neuropsychiatric disorders (odds ratio [OR]: 10.39, 95% CI: 9.89, 10.90).

**Conclusions:**

The accuracy of AUDIT in screening for alcohol disorders at a population-based level was high, but only identified one third of women with alcohol-related disorders when using a cut-off of six points criterion.

## Introduction

Alcohol-related diseases and disorders are a major global health challenge. Almost three million deaths per annum can be attributed to alcohol use, including but not restricted to associated medical disorders, interpersonal violence, and accidents (1). In Sweden, 86% of women have been assessed as current drinkers (1). Yet, drinking patterns differ across social and cultural contexts, as well as age (2). In the reproductive age, an increased susceptibility for development of substance-use disorders has been observed. About half of pregnant women report currently drinking just prior to pregnancy, with > 10% binge drinking (3, 4). Pre-conception binge drinking is associated with alcohol use during pregnancy (4). Therefore, adequate identification and support before/during/after pregnancy is crucial for maternal and neonatal health (5, 6), and is considered a top-priority for maternal health care in Sweden by the National Board of Health and Welfare. Psychiatric comorbidity often exists and women with neuropsychiatric disorders (e.g. ADHD) are at risk for developing substance use disorders (7). Substance use treatments encompass combined behavioral and pharmacological treatments (8).

Prenatal alcohol exposure is associated with adverse pregnancy outcomes. Such outcomes encompass birth defects, neurodevelopmental disorders, and fetal alcohol spectrum disorders, with differing degrees of severity and long-term consequences (9). No safe lower threshold has been established for alcohol consumption and totally refraining from alcohol consumption during pregnancy is advocated (10, 11). As a preventable cause, identification of alcohol consumption during pregnancy is essential for providing accurate information, treatment, counselling for the woman and her partner (10, 12). Furthermore, substance abuse affects parenting and care during childhood. Prenatal alcohol screening is important for implementing support measures and promoting social integration for the whole family (6, 13). Various alcohol use screening instruments, including questionnaires and biomarkers, have been proposed and are currently used in differing health care settings and populations (8, 12, 14). One of these screening instruments, proposed by the World Health Organization, is the Alcohol Use Disorders Identification Test (AUDIT), assessing frequency, level, and drinking behavior (15). Alcohol screening with the AUDIT is non-invasive and well-tolerated, providing opportunities for brief intervention or further referral. The AUDIT questionnaire has been validated for the pregnant population, but with a varying sensitivity ranging from 7 to 87% and a specificity of 86–100% (12). It has also been considered as non-reliable for identifying alcohol disorders in low risk use populations (16). As self-reported alcohol consumption often underestimates actual use, various alternative strategies have been proposed. Alcohol-specific biomarkers during pregnancy were compared with the self-reported AUDIT in a Swedish cohort (*n* = 2,264). Although 12% of women reported drinking during pregnancy, plasma biomarkers for alcohol disorders were negative during pregnancy. This indicates that a high consumption was needed for detection by these markers (17). In contrast, self-reported prenatal alcohol consumption, when compared with biomarkers in fetal meconium, have found a great discrepancy in rates of alcohol use during pregnancy (4.5% self-report vs. 18.9% identified by biomarker). This indicates a substantial underreporting (18). Furthermore, this is supported by a Swedish cross-sectional study that found a prevalence of hazardous alcohol use at the antenatal screening of 15.7% when using an anonymous self-report, compared with 5.1% if a non-anonymous questionnaire was used (19).

Nordic countries have a long-standing tradition of using population-based data for monitoring healthcare and research. *The Swedish Pregnancy Register* is a National Quality Register established in 2013 (20). In addition to pregnancy and birth-specific outcomes, it provides data on pre-pregnancy health, lifestyle and socioeconomic factors, including alcohol consumption during the year preceding pregnancy measured by the AUDIT questionnaire. The AUDIT provides non-anonymous information on self-reported alcohol use for a large pregnant population.

Increased use of data from quality registers in decision-making, political initiatives, and research requires appropriate investigation and the validation of entered data. Internal validity of maternal health care data in the *Swedish Pregnancy Register* was assessed by a cross-sectional study in 2014, demonstrating a good to excellent degree of coverage and agreement with data in maternal health care records. The registered AUDIT scores were identical for both sources in 96.6% of the women (21). However, an external validation of AUDIT’s ability to determine hazardous alcohol use before or during pregnancy has not been done at a population-based level. An alternative to manually cross-checking entered data in the register against clinical information or biomarkers, on a large scale, is combining register data from different resources on an individual level. Therefore, the aim of this study was to assess the external validity of AUDIT screening at prenatal care as an indicator of alcohol-addiction disorders recorded by diagnostic codes in national health registers. A secondary aim was to characterize women with false negative and false positive classification history of alcohol-related disorders, using an AUDIT cut-off score of six points.

## Methods

From the *Swedish Pregnancy Register* (20) information on 739,735 births from 2014 to 2020 was linked to the *Swedish National Patient Register* (22) and the *Swedish Prescribed Drug Register* through personal identification numbers. In the *Swedish Pregnancy Register,* clinical information is prospectively collected from standardized prenatal, obstetrical and neonatal medical records, or manually entered by midwives in primary maternity health care. Manually entered data for the register includes the score from the AUDIT questionnaire, recorded at the first antenatal visit; more precisely this should reflect alcohol consumption during the past year and not only ongoing consumption. The registered AUDIT score was categorized as AUDIT 0–5 (low risk use), AUDIT 6–13 (high risk use), AUDIT 14–40 (very high-risk use), or no AUDIT information. No information could represent the question was not asked, the woman did not answer, or that linkage with birth records was not possible due to administrative reasons (*n* = 43,257). For the purposes of the present study, AUDIT was also used as a dichotomized variable where scores ≥ 6 points indicated hazardous use and < 6 non-hazardous use (reference group), according to the proposed cut-off points for women in the Swedish setting (23). In a sub-analysis, use of a cut-off of ≥ 4 points for hazardous use was explored, as this has been proposed for other populations to increase sensitivity (24). Maternal characteristics registered at first visit included: country of birth (Sweden, other), maternal education (≤ 9, 10–12, >12 years), living condition (single mother household vs. cohabitant, also including other family situations such as extended family), smoking at first antenatal visit (yes regardless of quantity, no), and parity (nulliparous, parous). These data were based on midwife-reported information in electronic birth records. Prior psychiatric care (yes, no) could indicate a wide range of psychological treatments for psychiatric disorders. Age at delivery was grouped into ≤ 18, 19–34, ≥ 35 years.

Alcohol-related disorders were defined by the diagnostic code F10 (disorders related to or resulting from abuse or misuse of alcohol). Data were obtained from in- and outpatient visits recorded in the *Swedish National Patient Register* 2003–2020 and/or the Anatomical Therapeutic Chemical (ATC) code N07BB (drugs used in alcohol dependence) from the *Swedish Prescribed Drug Register* 2006–2020. Alcohol disorders were defined as overall (registered anytime during the above defined time period), pre-pregnancy (restricted to during 1 year before first antenatal visit), or post-pregnancy (registered after birth). Neuropsychiatric disorders were defined by the International Classification of Disease version 10 (ICD-10) code F90-98 in the *Swedish National Patient Register* 2003–2020 and/or the ATC code N06B (psychostimulants) from the *Swedish Prescribed Drug Register* 2006–2020.

### Statistical analyses

The diagnostic discriminatory and predictive properties of AUDIT were examined by calculating: sensitivity, specificity, accuracy, positive predictive value (PPV), negative predictive value (NPV), positive likelihood ratio (LR+), and negative likelihood ratio (LR-) for AUDIT score ≥ 6 (the test) for the outcome alcohol disorders within 1-year preceding conception and up to 6 years post-pregnancy. LR+ (rate of true positive/false positive) expresses the probability of a given test result (AUDIT ≥ 6) in a patient for an alcohol disorder compared with a patient without alcohol disorder. LR- (rate of false negative/true negative) is the likelihood for a negative test result in an individual with alcohol disorder compared without this disorder. To determine an optimal cut-off, a receiver operating characteristics (ROC) curve of AUDIT points at a continuous scale for pre-pregnancy alcohol disorders was constructed, and Youden’s index ((sensitivity +specificity) –1) was calculated to select an optimal cut-off combining sensitivity and specificity. Accuracy was the proportion (%) of correctly classified subjects (either true positive or true negative). Women with mismatched information in the register (false negative AUDIT and false positive AUDIT) were characterized according to maternal characteristics. Associations between characteristics and a matching category were explored by unadjusted multinominal logistic regression models expressed as odds ratios (ORs) with 95% Confidence Intervals (CIs). Women with matched information in all registers (true negative and true positive) was the reference category. To ensure non-dependency of analysis, only the first birth registered in the *Swedish Pregnancy Register* for woman with AUDIT points registered (*N* = 544,007) was included in the analysis (*n* = 454,027). Statistical analyses were performed using SPSS software for Mac, version 25 (IBM Corp., Armonk, NY, USA) and calculations of diagnostic accuracy based on aggregated numbers were performed by the open software program ©MedCalc Software Ltd (accessed 2023).

## Patient and public involvement

There was no patient or public involvement in this study.

## Results

Out of 739,735 singleton births, pre- or post-pregnancy alcohol-related disorder was recorded in 3.1% and 25,770 (3.5%) had an AUDIT point ≥ 6 points. The rate of registered alcohol-related disorder pre- or post-pregnancy increased with the AUDIT category, from 2.7% in AUDIT 0–5 points (low risk use) to 40.6% in the group with AUDIT 14–40 points (very high-risk use). For women without AUDIT information, the rate of alcohol disorders overall was 3.0% (Table 1). The diagnostic accuracy of the AUDIT ≥ 6 points for detecting a related alcohol disorder during 1 year before pregnancy was 95.7% (95% CI: 95.7, 95.8), with a high *positive likelihood ratio* of 8.03 (95% CI: 7.5, 8.6). Sensitivity using the cut-off of 6 points or more for detecting a pre-pregnancy alcohol-related disorder was 33.0% (95% CI: 30.9, 35.1), the sensitivity was even lower for detecting alcohol disorders post-pregnancy (24.9%, 95% CI: 23.3, 26.4). In contrast, the specificity of the AUDIT test was high, both pre- and post-pregnancy (95.9%, 95% CI: 95.9, 96.0) (Table 2).

**Table 1:** AUDIT categories and alcohol disorders before or after pregnancy (*N* = 739,735).

AUDIT points (rate%)	Alcohol disorder ever *n* = 22,988 (3.1%)	Pre-pregnancy^a^ *n* = 2,418 (0.3%)	Post-pregnancy *n* = 4,019 (0.5%)
AUDIT 0–5 (79.5%)	16,140 (2.7)	1,273 (0.2)	2,344 (0.4)
AUDIT 6–13 (3.3%)	2,420 (10.0)	384 (1.6)	497 (2.1)
AUDIT 14–40 (0.2%)	623 (40.6)	242 (15.8)	278 (18.1)
No AUDIT (17.0%)	3,805 (3.0)	519 (0.4)	900 (0.7)

Alcohol disorder refers to the diagnostic code F10 (ICD-10) from in- or outpatient national health registers (2003–2020) and/or ATC code N07BB from the The Prescribed Drug Register (2006–2020).

AUDIT information was retrieved from manually entered information from pregnancies recorded in the Swedish pregnancy register 2014–2020.

a Registered one year before first antenatal visit.

**Table 2:** Diagnostic accuracy of AUDIT ≥ 6 points (*n* = 25,770) according to alcohol disorders recorded before or after pregnancy.

Alcohol disorder	TP	FP	TN	FN	Sensitivity	Specificity	PPV	NPV	LR+	LR-
	*n* =	*n* =	*n* =	*n* =	% (95% CI)	% (95% CI)	% (95% CI)	% (95% CI)	% ( 95% CI)	% (95% CI)
Pre-pregnancy	626	25,144	587,065	1,273	33.0 (30.9, 35.1)	95.9 (95.8, 95.9)	2.4 (2.3, 2.6)	99.8 (99.8, 99.8)	8.03 (7.5, 8.6)	0.70 (0.7, 0.7)
Post-pregnancy	775	24,995	585,994	2,344	24.9 (23.3, 26.4)	95.9 (95.9, 96.0)	3.0 (2.8, 3.2)	99.6 (99.6, 99.6)	6.07 (5.7, 6.5)	0.78 (0.8, 0.8)

TP: true positive; FP: false positive; TN: true negative; FN: false negative; CI: confidence interval.

Sensitivity, specificity, positive and negative predictive value expressed as percentages with 95% CI. Likelihood ratio calculated as odds ratios with 95% CI.

MedCalc Software Ltd. Diagnostic test evaluation calculator. https://www.medcalc.org/calc/diagnostic_test.php (Version 20.105); accessed April 5, 2022.

Younger age, Swedish origin, single mother household, smoking, nulliparity, and prior psychiatric disorders were associated with an increased likelihood of a false positive result in AUDIT screening for alcohol-related diagnosis or treatment. These characteristics were consistent and more pronounced for a false negative result where prior psychiatric care increased the odds four-fold (OR: 4.23, 95% CI: 4.07,4.40). Neuropsychiatric disorders carried an over 10-fold increased OR for having a false negative result in AUDIT screening (Table 3). The ROC curve (Supplementary Fig. 1) indicated a maximal Youden index of 0.322 at AUDIT ≥ 4.5 points. If a cut-off of ≥ 4 points for AUDIT was used, the sensitivity for detecting an alcohol disorder 1 year before pregnancy increased from 33 to 47%, but specificity was lowered, resulting in a reduced test accuracy of 84% (95% CI: 84.3, 84.4) (Supplementary Table 1).

**Table 3:** Maternal characteristics according to diagnostic accuracy of the AUDIT score registered at the first pregnancy (*N* = 454,027).

	Match			Mis-match			
	Missing *N*	Rate %	Reference *n* = 424,385	False positive *n* = 17,751	OR (95% CI)	False negative *n* = 11,891	OR (95% CI)
Age (years)	-	-	-	-	-	-	-
≤ 18	-	0.2	677	49	2.75 (2.05, 3.68)	40	3.51 (2.54, 4.84)
19–34	-	75.9	319,337	14,955	1.78 (1.71, 1.85)	10,092	1.88 (1.78, 1.97)
≥ 35	-	23.9	104,371	2,747	1.0	1,759	1.0
Swedish origin	31,634	72.5	280,500	15,202	4.56 (4.31, 4.82)	10,718	3.87 (3.64, 4.12)
Maternal education (years)	64,561	-	-	-	-	-	-
≤ 9	-	8.2	29,636	880	0.82 (0.76, 0.88)	1,470	2.98 (2.80, 3.18)
10–12	-	38.5	136,590	7,221	1.45 (1.40, 1.50)	6,175	2.72 (2.61, 2.84)
> 12	-	53.3	197,042	7,177	1.0	3,275	1.0
Single mother	34,409	2.2	7,991	572	1.73 (1.58, 1.88)	515	2.38 (2.18, 2.61)
Smoking	23,878	4.6	16,337	1,599	2.50 (2.37, 2.64)	1,985	5.09 (4.84, 5.36)
Nulliparous	22	46.1	188,984	14,815	6.29 (6.04, 6.55)	5,652	1.13 (1.09, 1.17)
Prior psychiatric care	25,485	14.5	53,385	4,444	2.36 (2.28, 2.44)	4,402	4.23 (4.07, 4.40)
Neuropsychiatric disorder	-	3.1	10,416	1,006	2.39 (2.23, 2.55)	2,464	10.39 (9.89, 10.90)

Match= true positive and true negative in screening with an AUDIT cut-off ≥ 6 points for alcohol disorders ever was used as the reference category.

Mis-match= false positive and false negative in screening with an AUDIT cut-off ≥ 6 points for alcohol disorders ever.

Behavioral disorder: ICD 10 F90–98 before or after or ATC code.

Associations between maternal characteristics and being mis-matched was investigated with multinominal logistic regression with match category as the reference. Associations are reported as unadjusted odds ratios (ORs) with 95% Confidence Intervals (CIs).

## Discussion

At population-level antenatal screening, AUDIT demonstrates a high accuracy and a positive LR of alcohol-related disorders when screening positive. Higher points on AUDIT were associated with higher rates of alcohol disorders before and after pregnancy. However, an AUDIT with a cut-off of 6 points identified only about one-third of women with alcohol-related disorders the year preceding pregnancy, indicating that the threshold of 6 points is too high or that non-anonymous self-reporting is not optimal. The NPV of the test (i.e. the ability of AUDIT to rule out the disorder) was high, driven by a greater specificity and low prevalence of alcohol-related disorders treated in specialized care. Women with false negative results were most strongly characterized by younger age, lower education level, smoking, and a history of psychiatric disorders. Nulliparity and being Swedish-born most strongly increased the likelihood of being false positive at screening. This could represent a risk-use of alcohol but not an alcohol dependency yielding a diagnosis or treatment.

In this population-based study, 3.5% had an AUDIT point ≥ 6 at the first antenatal visit, but this only identified a third of women with a registered alcohol disorder in the year preceding pregnancy. This could be consistent with a successful treatment of alcohol-dependency rather than a false negative in screening, although the AUDIT should reflect alcohol consumption during the past year and not only ongoing consumption. The prevalence of alcohol use during pregnancy is influenced by collection methods and higher rates have been reported in differing populations and time-periods (17, 18, 25, 26). For example, anonymous reporting increased the rate of positive screening in a similar setting with AUDIT from 5.1 to 15.7% (19). In the USA, currently 11.5% of pregnant women reported drinking using non-anonymous self-report by telephone interviews. Drinking and binge drinking occurred more frequently among women ≥ 35 years and unmarried women (27). We found that single mothers had a more than two-fold increased likelihood of screening false negative with AUDIT for alcohol-related disorders. Other socio-demographic factors associated with misclassification of the AUDIT cut-off ≥ 6 was a low education level and being born in Sweden. Previous psychiatric care was associated with a false negative result, that is the AUDIT did not capture alcohol-related disorders 1-year pre-pregnancy well in women with a history of psychiatric care. More specifically, behavioral disorders including neuropsychiatric disorders increased the odds of a false negative more than 10-fold. This corresponds to a group of at-risk women vulnerable for a wide range of reproductive complications, such as repeat abortions, teenage pregnancies, and preterm birth (28, 29). Furthermore, smoking and illicit-drug use during pregnancy are more commonly reported in women with neuropsychiatric disorders (7, 28). These factors also increased the likelihood of screening false positive or false negative with AUDIT. The psycho-social burden of alcohol use disorders is well-established and additional support in prenatal and postpartum care for these women is certainly warranted (28).

As self-reported alcohol consumption could cause underestimation of true use, biomarkers could serve as a supporting complement by confirming high-risk alcohol use (26). May et al. found that 3.1–6.8% screened positive on biomarkers, but not AUDIT using a cut-off of 4 points. In contrast, for a low risk use population, alcohol biomarkers could not identify any alcohol use. An optimal biomarker for routine, large-scale alcohol screening among pregnant women has yet to be proposed (17). Nulliparity increased the odds of being false-positive (i.e. identified risk-consumption prior to pregnancy but no alcohol disorder). The AUDIT test has been shown to be a non-optimal screening tool for alcohol disorders among women and low-risk users (16). In our study, a diagnosed alcohol use disorder corresponded with substance use requiring medical care or treatment. An AUDIT score of 6–13 points is considered risk-use but not equal to alcohol dependency. When a cut-off of ≥ 4 points for the AUDIT was used, the sensitivity for detecting an alcohol disorder 1 year before pregnancy increased from 33 to 47%, but reduced the proportion of correctly classified individuals of the test from 96 to 84%. The clinical use of AUDIT in maternal health care services is to identify women with risk of alcohol use during pregnancy and to offer counselling when necessary. For the diagnostic evaluation, we did not restrict our analysis to the year preceding pregnancy but also investigated the ability of AUDIT (with a cut-off of ≥ 6) to correctly classify women with an alcohol disorder post-pregnancy. Hazardous alcohol consumption postpartum affects parenting and childhood care (6). Prenatal alcohol screening with AUDIT could also help identify women and families in need of social support postpartum also.

We acknowledge there are certain limitations to the present study. To assess the discriminatory ability of AUDIT (the test) to identify alcohol-related disorders, we defined having an in- or outpatient diagnosis of alcohol-related disorders or dispensed medication to treat alcohol disorders pre- or post-pregnancy. This can be considered unreliable and misclassification of alcohol disorders cannot be ruled out, even though the proportion of valid diagnoses in the register is considered high for severe diseases, including psychiatric disorders (22). According to the Public Health Agency of Sweden, 16–32% of adults had a risky consumption of alcohol in 2021 (30). This indicates an underreporting at prenatal care as only 3.5% of women had an AUDIT score ≥ 6 in our cohort. The AUDIT registered at maternal health care should reflect alcohol consumption during the past year and not only ongoing consumption, but we do not know if women answer the test to reflect current use. We cannot rule out the fact that the low sensitivity observed to identify alcohol disorders pre-pregnancy is a consequence of treatment of an already identified high-risk group. Although, AUDIT had a high negative predicted value, only one in four women with alcohol disorders postpartum were identified using a cut-off ≥ 6 points.

In conclusion, we found that the accuracy of the AUDIT of ≥ 6 points for alcohol disorders at a population-based level was high, but the test sensitivity was only 33%. A false negative result in AUDIT was associated with younger age, lower education-level, being Swedish-born, and nulliparity. Vulnerable groups include women with prior psychiatric care and neuropsychiatric disorders (including ADHD). This merits special attention when AUDIT screening is performed and evaluated in maternal healthcare.

## Strengths and limitations

- Population-based study
- Prospectively collected information of alcohol-use according to the AUDIT
- External validation of information in a National register used for quality and research

## Supplementary Material





## Data Availability

Data for this research project are available from the National Board of Health and Welfare in Sweden and the Swedish Pregnancy Register, which does not permit data-sharing according to the Swedish Secrecy Act. Women have the ability to opt-out of the Swedish Pregnancy Register.
