# Supplementary material for: Validity of prenatal AUDIT screening for alcohol disorders – a Nationwide Swedish register study
Source: Ups J Med Sci. 2024 Nov 22;129:10.48101/ujms.v129.10770. doi: 10.48101/ujms.v129.10770 (PMC11650420; doi:10.48101/ujms.v129.10770)
Supplement: Supplementary file 1 [file UJMS-129-10770-s1.pdf]

Table S1. Diagnostic accuracy of AUDIT ≥4 points according to alcohol disorders recorded one year before pregnancy.

	AUDIT≥4										
	**TP** n=	**FP** n=	**TN** n=	**FN** n=	**Sensitivity** % (95 % CI)	**Specificity** % (95 % CI)	**PPV** % (95 % CI)	**NPV** % (95 % CI)	**Accuracy** % (95 % CI)	**LR+** OR (95 % CI)	**LR-** OR (95 % CI)
**Alcohol disorder°**	895	95185	517024	1004	47.1 (44.9, 49.4)	84.5 (84.4,84.5)	0.93 (0.9,1.0)	99.8 (99.8,99.8)	84.3 (84.3,84.4)	3.03 (2.89,3.18)	0.63 (0.60,0.65)

°Registered one year before first antenatal visit

Sensitivity, specificity, positive and negative predictive value, accuracy is expressed as percentages with 95 % confidence interval (CI)

MedCalc Software Ltd. Diagnostic test evaluation calculator. https://www.medcalc.org/calc/diagnostic_test.php (Version 20.105; accessed Dec, 2022
