# Supplementary material for: Validity of prenatal AUDIT screening for alcohol disorders – a Nationwide Swedish register study
Source: Ups J Med Sci. 2024 Nov 22;129:10.48101/ujms.v129.10770. doi: 10.48101/ujms.v129.10770 (PMC11650420; doi:10.48101/ujms.v129.10770)
Supplement: Supplementary file 2 [file UJMS-129-10770-s2.pptx]

## Slide 1
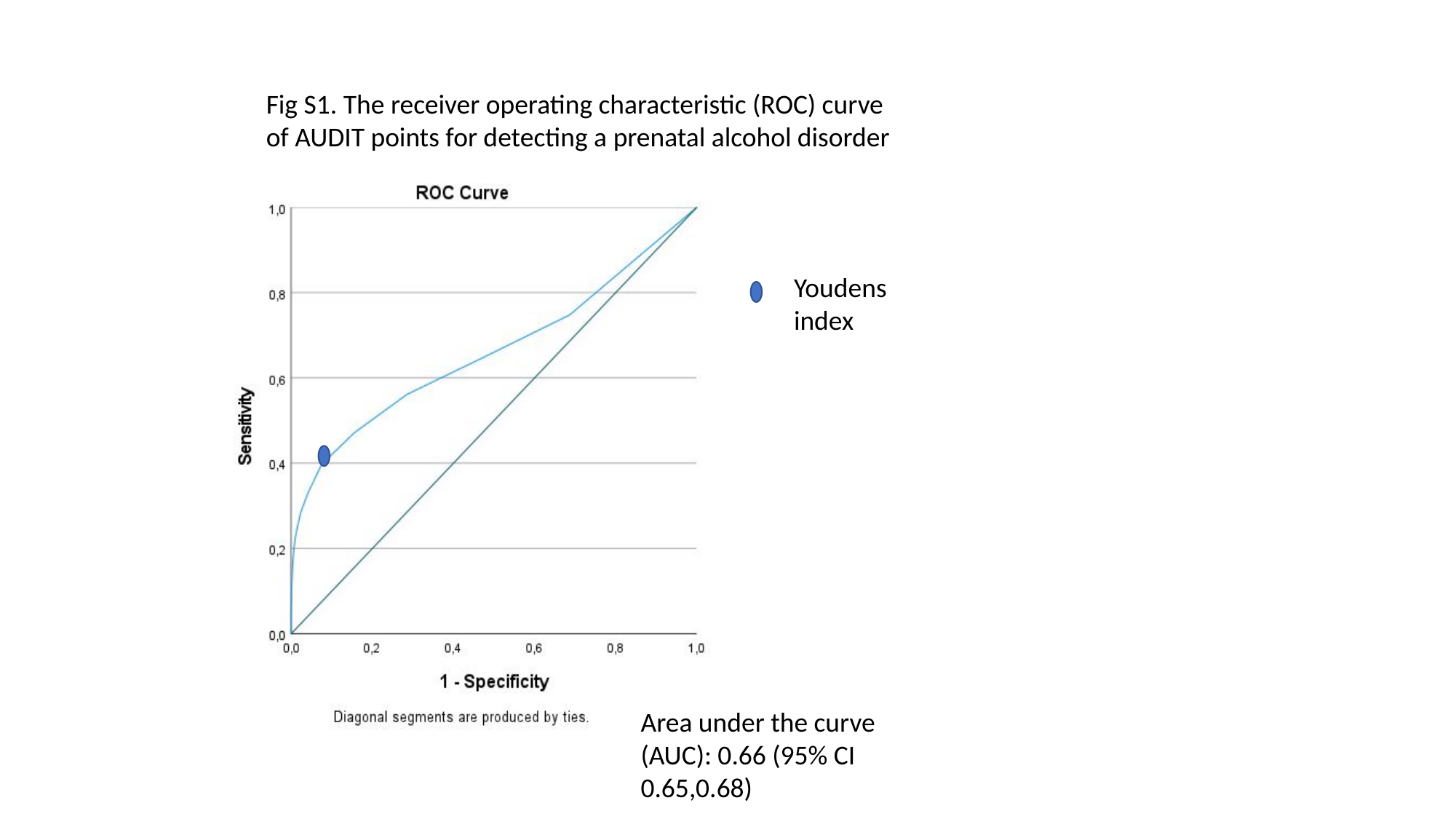

Fig S1. The receiver operating characteristic (ROC) curve of AUDIT points for detecting a prenatal alcohol disorder
Youdens index
Area under the curve (AUC): 0.66 (95% CI 0.65,0.68)
